# Changes in body mass index by age, gender, and socio-economic status among a cohort of Norwegian men and women (1990–2001)

**DOI:** 10.1186/1471-2458-7-269

**Published:** 2007-09-30

**Authors:** Deborah L Reas, Jan F Nygård, Elisabeth Svensson, Tom Sørensen, Inger Sandanger

**Affiliations:** 1 Institute of Psychiatry, Faculty of Medicine, University of Oslo, Norway; 2 Health Region East, Health Services Research Center, Akershus University Hospital, Oslo, Norway; 3 Health Region East, Health Services Research Center, University of Oslo, Norway

## Abstract

**Background:**

Consistent with global trends, the prevalence of obesity is increasing among Norwegian adults. This study aimed to investigate individual trends in BMI (kg/m^2^) by age, gender, and socio-economic status over an 11-year period.

**Methods:**

A cohort of 1169 adults (n = 581 men; n = 588 women) self-reported BMI during a general health interview twice administered in two regions in Norway.

**Results:**

Average BMI increased significantly from 23.7 (SD = 3.4) to 25.4 (SD = 3.8), with equivalent increases for both genders. Proportion of obesity (BMI ≥ 30) increased from 4% to 11% for women and 5% to 13% for men. Of those already classified as overweight or obese in 1990, 68% had gained additional weight 10 years later, by an average increase of 2.6 BMI units. The greatest amount of weight gain occurred for the youngest adults (aged 20–29 years). Age-adjusted general linear models revealed that in 1990, women with a lower level of education had a significantly greater BMI than more educated women. In both 1990 and 2001, rural men with the highest level of household income had a greater BMI than rural men earning less income. Weight gain occurred across all education and income brackets, with no differential associations between SES strata and changes in BMI for either gender or region.

**Conclusion:**

Results demonstrated significant yet gender-equivalent increases in BMI over an 11-year period within this cohort of Norwegian adults. Whereas socio-economic status exerted minimal influence on changes in BMI over time, young adulthood appeared to be a critical time period at which accelerated weight gain occurred.

## Background

In line with trends worldwide, epidemiological studies have documented an increasing prevalence of obesity in Scandinavia. Data from population-based health surveys administered in the mid-80 and mid-90's showed rising obesity rates among Norwegian adults from 6.7% to 15.5% for men and from 11% to 21% for women [1]. This trend toward increasing body mass index (BMI) also parallels reports from neighboring Sweden [2] and Denmark [3].

The underlying determinants of a shifting BMI distribution are inarguably complex, but are indicative of unfavorable societal and environmental conditions which promote inactivity, excessive energy intake, and malnutrition [4]. In industrialized nations, an inverse association between BMI and low socio-economic status (SES) has long been recognized as a public health concern [5]. Elucidating and eliminating disparities in obesity among socio-economic strata represents an international priority [6]. Education and income gradients are thought to affect obesity-related health behaviors, such as television viewing [7], work and leisure sitting time [8], food purchasing behavior [9], access to healthy foods [10], access to physical activity facilities [11], and a host of neighborhood characteristics such as crime and walkability [12].

Although a recent meta-analysis of the effects of SES on body mass index found consistent support for an inverse association between weight gain and occupational type, only equivocal evidence was found for level of education, and inconsistent support existed for income [13]. Data from the US National Health and Nutrition Examination have demonstrated disappearing economic disparities in obesity rates over the past 30 years, suggesting that a prolonged and progressive positive energy balance has extended across different population segments [14]. The authors argued that an increasingly pervasive obesogenic environment may eventually work to narrow social gaps in BMI distribution. In Norway, studies of socio-economic strata and BMI have focused largely on children and adolescents and have yielded mixed findings [15-17]. Understanding longitudinal shifts in overweight and obesity among different segments of society may prove valuable in shaping public health policy, lend insight into the interplay between health and the environment, and may help guide our efforts at obesity management and ultimately, prevention. The present study examined changes in BMI according to age, gender, and socio-economic status over an 11-year period in a cohort of Norwegian adults.

## Method

### Participants

Participants were recruited for the OsLof study, which is an ongoing and prospective population-based survey initiated in 1990 and designed to examine general health and mental health within two geographically diverse areas of Norway. The cohort of individuals participating at both timepoints (N = 1300) was selected for the present study to examine individual trends in BMI (kg/m^2^) according to age, gender, and socio-economic status. After excluding N = 131 participants with missing or invalid height or weight data, the final sample was comprised of N = 1169 adults. Additional details regarding the survey are presented elsewhere [18]. This study was approved by the Norwegian Data Inspectorate and written informed consent was obtained from all participants prior to enrollment.

### Study procedures

Height and weight data were collected via self-report during a 2-hour fully structured interview, which included the Composite Diagnostic Interview [19] and the Hopkins Symptom Checklist-25 [20], as well as questionnaire items to assess demographics and mental and physical health (e.g., substance abuse, negative life events, social support, service utilization, attitudes towards mental health services, etc.). The routines for data collection were standardized and repeated similarly in 1990 and 2001 by trained interviewers.

Body mass index (BMI) was calculated using the formula (kg/m^2^) and defined as weight (kg) divided by height (m) squared. Consistent with established criteria, "overweight" was defined as a BMI of 25–29.9 kg/m^2 ^and "obesity" was defined as a BMI of ≥ 30. "Weight gain" was defined as an increase of a minimum of 1 kg (2.2 pounds) in addition to a positive change in BMI. "Weight loss" was defined as a minimum loss of 1 kg (2.2 pounds) plus a negative change in BMI. Highest level of education achieved was classified at both timepoints as *low *(9 years; compulsory education), *middle *(10–12 years; upper secondary school), and *high *(13 or more; college or university). Income level cut-offs were determined statistically to create approximately three equal groups at each timepoint (low, middle, and high). Analyses were based on total household income and individuals reporting absolutely zero income were excluded. Due to significant differences in level of income and cost of living between participants living in Lofoten (rural area in Northern Norway) and Oslo (urban), income levels were stratified by region. For rural residents, income levels in 1990 and 2000–01 were classified as *low *(< 199,999 NOK and < 228,665 NOK), *middle *(≥ 200,000 to 299,999 and ≥ 228,666 to 419,999 NOK), and *high *(≥ 300,000 NOK and ≥ 420,000 NOK). For urban residents, income levels in 1990 and 2000–01 were classified as *low *(< 300,000 NOK and < 399,999 NOK), *middle *(≥300,000 to 399,999 NOK and ≥ 400,000 to 649,999 NOK), and *high *(≥ 400,000 NOK and ≥ 650,000 NOK).

### Data analysis

Body weight in kilograms (kg) and BMI were described at each timepoint and longitudinal changes were assessed, as were proportions overweight or obesity according to gender and age specifications. Bivariate correlations between age and changes in BMI were directly examined by computing a Pearson's correlation coefficient and chi-squares. To test the effects of educational level and level of income on BMI at each timepoint, gender-specific general linear models ANOVAs were conducted, with age entered as a covariate. Statistical analyses were performed using the Statistical Package for Social Sciences (SPSS) version 13.0.

## Results

### Sample characteristics

The present sample consisted of 1169 adult men and women with height (cm) and weight (kg) data collected at two timepoints (1990–91 and 2000–01). The gender distribution was 588 (50.3%) women and 581 (49.7%) men. The average age for the sample in 1990 was 40.4 years (± 13.7; range 19–84 years). A total of 584 (50.0%) respondents resided in Oslo and 585 (50.0%) were from Lofoten. A both timepoints, the majority (over 70%) were married or cohabitating. At baseline, 30.9% had a compulsory education, 36.9% had finished upper secondary school, and 32.2% had received a university or college education. Corresponding educational data at follow-up were: 25.6%, 35.1%, and 39.3%, respectively. In 2001, the average household income in Oslo was NOK 553,771 (approx. 85,000 USD) compared to NOK 330,459 in rural Lofoten (approx. 51,000 USD).

### Body mass index and weight data

**Figure 1:**
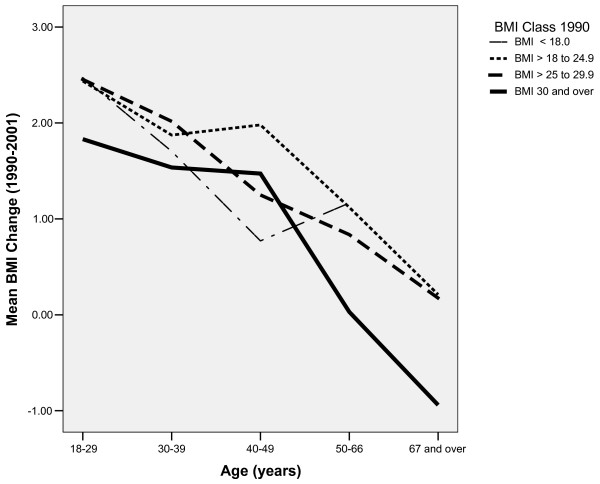
Mean change in BMI grouped by age and BMI status.

**Figure 2:**
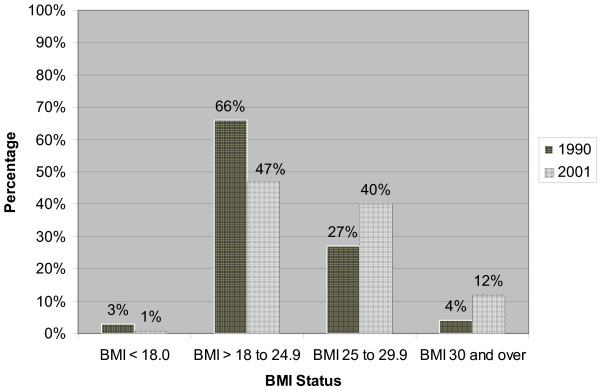
BMI status at 1990 and 2001.

A summary of mean weight (kg), BMI, and changes in BMI is presented in Table 1. In 1990, the mean BMI for the overall sample was 23.7 (SD = 3.4), increasing to 25.4 (SD = 3.8) by the year 2001. Men and women underwent equivalent increases in BMI (F = 1.6, p = 0.19). Our results show that the greatest changes in weight and BMI occurred for the younger age groups (see Table 1), and a Pearson's correlation between age and BMI change was negative and significant (r = -0.30, p < 0.001 for men and r = -0.25, p < 0.001 for women). As shown in Figure 1, greater weight gains among the youngest occurred across all four 1990 BMI classes (underweight, normal weight, overweight, and obese). The overall distribution of BMI class for 1990 and 2001 is illustrated by Figure 2 and Table 2 provides a breakdown of BMI class according age and gender. These data indicated an overall increase in proportion overweight from 27.1% to 40.2%. Proportion of obesity increased from 4.3% to 11.7%, while the proportion of individuals with a normal weight decreased from 66.0% to 47.0%.

**Table 1:** Mean weight (kg) and BMI by age and gender

		**Mean (SD)**			**Mean (SD)**		**Mean change (SD)**	
		1990			2001		1990–2001¶	

	N	Weight (kg)	BMI		Weight (kg)	BMI	Weight (kg)	BMI
Men				Men				
20–29	123	76.0 (10.9)	23.6 (2.9)	20–29	84.0 (13.9)	26.1 (3.9)	8.0 (8.6)	2.5 (2.5)
30–39	176	80.0 (11.2)	24.4 (2.9)	30–39	85.9 (12.6)	26.2 (3.3)	5.9 (5.5)	1.8 (1.7)
40–49	135	81.5 (10.9)	25.2 (2.7)	40–49	86.0 (12.3)	26.6 (3.2)	4.5 (6.1)	1.4 (1.9)
50–66	109	80.1 (10.4)	25.5 (3.4)	50–66	82.9 (11.9)	26.4 (3.8)	2.8 (7.2)	0.9 (2.3)
67–79	38	77.7 (11.4)	25.4 (3.4)	67–79	78.6 (11.5)	25.7 (3.6)	0.9 (6.7)	0.3 (2.1)
								
Total	581	79.4 (11.1)	24.7 (3.0)	Total	84.5 (12.7)	26.3 (3.5)	5.1 (7.1)	1.6 (2.2)
								
Women				Women				
20–29	148	61.7 (11.3)	21.8 (3.5)	20–29	68.6 (13.0)	24.2 (4.0)	6.9 (7.5)	2.4 (2.7)
30–39	189	61.5 (8.7)	22.1 (2.7)	30–39	67.0 (10.4)	24.0 (3.4)	5.5 (5.4)	2.0 (1.9)
40–49	123	64.7 (10.7)	23.5 (3.7)	40–49	70.0 (11.9)	25.5 (4.3)	5.3 (7.1)	2.0 (2.6)
50–66	104	65.5 (12.2)	24.3 (4.0)	50–66	67.7 (12.0)	25.1 (4.0)	2.2 (6.3)	0.9 (2.3)
67–79	24	68.0 (12.4)	25.4 (3.8)	67–79	67.0 (12.0)	25.1 (3.7)	-1.0 (4.5)	-0.3 (1.7)
								
Total	588	63.2 (10.7)	22.8 (3.6)	Total	68.1 (11.8)	24.6 (3.9)	5.0 (6.8)	1.8 (2.4)

BMI, body mass index; kg, kilograms.

¶ Age at year 2001 used in formula.

**Table 2:** Percentage overweight and obese by age and gender (1990 and 2001)

		**1990**			**2001**	
	N	% Overweight (BMI ≥ 25 to 30)	% Obese (BMI ≥ 30)		% Overweight (BMI ≥ 25 to 30)	% Obese (BMI ≥ 30)

Men				Men		
18–29	123	20.3	4.1	18–29	44.7	12.2
30–39	176	35.2	3.4	30–39	54.0	13.1
40–49	135	45.9	4.4	40–49	60.7	13.3
50–66	109	42.2	6.4	50–66	45.0	11.9
67–79	38	36.8	10.5	67–79	44.7	10.5
						
Total	581	36	4.8	Total	51.3	12.6
						
Women				Women		
18–29	148	11.5	4.0	18–29	23.6	9.5
30–39	189	13.2	0.5	30–39	26.5	7.9
40–49	123	20.3	5.7	40–49	35.0	16.3
50–66	104	30.8	6.7	50–66	37.5	10.6
67–79	24	37.5	8.3	67–79	20.8	16.7
						
Total	588	18.4	3.7	Total	29.3	10.9

BMI, body mass index.

In 1990, chi-squares were *x*^2 ^= 30.2 (p < 0.01) and *x*^2 ^= 48.8 (p = 0.01) for men and women.

In 2001, chi-squares were *x*^2 ^= 13.3 (p = 0.10) and *x*^2 ^= 22.1 (p < 0.05) for men and women.

Over the 11-year period, 18.0% lost weight, 76.7% gained weight, and 5.2% reported no weight change. No gender differences existed in the proportion who gained, lost, or remained the same weight over time (*x*^2 ^= 0.81, p = 0.66). Individuals who lost weight or remained weight stable were significantly older and had a higher BMI at baseline than those who gained weight (F = 32.4, p < 0.001; F = 19.2, p < 0.001). We examined weight course for the 367 individuals classified as obese or overweight in 1990, as they were deemed a natural "at-risk" subgroup. The majority (68.1%) had gained additional weight eleven years later. The average weight gain for these individuals was 7.8 kg (SD = 6.3), which was a statistically significant increase and equaled a BMI unit increase of 2.6 (SD = 2.1); t (249) = -20.2, p < 0.01).

Table 3 presents a summary of findings for socio-economic status and BMI. In 1990 only, women with a compulsory (i.e., less) education had a significantly higher BMI than more educated women. For men living in the rural area, BMI values for both 1990 and 2001 differed significantly by income level, such that higher earners had a significantly higher BMI than middle and low-income earners (F = 3.6, p < 0.05). No similar effects for income were found for women (rural or urban) or for men residing in Oslo. All levels of income and education demonstrated weight gain between 1990 and 2001, with no differential associations between the SES indicators and changes in BMI.

**Table 3:** Actual BMI and changes in BMI by level of income and education in 1990 and 2001

	**1990**		**2001**		**1990–2001**	
	BMI kg/m2		BMI kg/m2		BMI unit change¶	
	Mean (SE)	F (p-value)	Mean (SE)	F (p-value)	Mean (SE)	F (p-value)

**Men**						
Education						
Low	24.8 (0.24)		26.4 (0.31)		1.6 (0.18)	
Middle	24.6 (0.22)	0.27 (0.76)	26.4 (0.25)	0.35 (0.71)	1.6 (0.15)	0.10 (0.91)
High	24.6 (0.22)		26.1 (0.25)		1.5 (0.14)	
Income Level						
Urban						
Low	24.0 (0.30)		26.1 (0.38)		2.1 (0.22)	
Middle	24.5 (0.29)	1.5 (0.25)	26.3 (0.36)	0.44 (0.64)	1.7 (0.21)	0.79 (0.45)
High	24.8 (0.32)		26.7 (0.39)		1.9 (0.23)	
Rural						
Low	24.6 (0.31)		26.0 (0.36)		1.3 (0.21)	
Middle	24.4 (0.31)	6.3 (0.001)**	25.5 (0.36)	4.2 (0.02)*	0.98 (0.20)	0.80 (0.45)
High	26.2 (0.38)		27.1 (0.43)		0.98 (0.25)	
						
**Women**						
Education						
Low	23.5 (0.29)		25.1 (0.36)		1.8 (0.22)	
Middle	22.6 (0.23)	3.3 (0.03)*	24.5 (0.27)	1.4 (0.25)	1.8 (0.17)	0.04 (0.96)
High	22.4 (0.26)		24.4 (0.26)		1.8 (0.16)	
Income Level						
Urban						
Low	22.0 (0.26)		23.8 (0.31)		1.8 (0.21)	
Middle	22.3 (0.33)	0.21 (0.82)	23.9 (0.38)	0.45 (0.64)	1.6 (0.26)	0.67 (0.51)
High	22.2 (0.38)		24.3 (0.44)		2.1 (0.30)	
Rural						
Low	23.5 (0.39)		25.3 (0.55)		1.8 (0.26)	
Middle	23.6 (0.47)	0.24 (0.78)	25.1 (0.54)	0.14 (0.87)	1.5 (0.31)	0.29 (0.74)
High	23 1 (0 45)		25 0 (0 51)		1 8 (0 29)	

Age-controlled results shown. Estimated marginal means and standard errors are depicted. BMI, body mass index.

¶ Year 2001 education and income level used in formula; * significant at p ≤ 0.05 level; ** significant at p ≤ 0.01 level.

## Discussion

Our results demonstrated significant increases in BMI and prevalence of obesity within this cohort of Norwegian men and women across an 11-year period. The proportion of normal weight individuals fell from 66% to 47%, while percent overweight increased from 27% to 40% and percent obesity increased from 4% to 12%. Men and women underwent equivalent gains in BMI over time, and 76% of both genders gained weight between 1990 and 2001. Of those already classified as overweight or obese in 1990, 68% had gained additional weight ten years later.

Our age-related findings concur with prior studies signifying greater weight gains for younger adults. For example, a Norwegian study initiated in the mid-80s found that men and women aged 20–29 years gained an average of 7.9 kg and 7.3 kg across an 11-year period, which represented the greatest increases in their sample [1]. Similarly, a recent 22-year study of adults living in northern Norway demonstrated the greatest weight gains among the youngest age group [21]. National surveys in the United States, namely CARDIA and NHANES, have witnessed average gains among adults aged 20–40 years of approximately 1.8 to 2.0 pounds (0.8 to 1.0 kg) per year. Although our 2-point data collection precludes details regarding the exact course and speed of weight gain, men aged 20–29 years in our study averaged an annual weight gain of 1.7 pounds (0.8 kilos) and women aged 20–29 years averaged an annual weight gain of 1.5 pounds (0.7 kilos). Despite appearing modest, such annual increases accumulate if continued over protracted periods to increase risk for obesity.

Level of income exerted a differential influence on BMI for men residing in the rural region. Specifically, results showed that rural men with the highest household income had significantly greater BMI at both timepoints, which seemingly runs counter to previous studies from industrialized nations demonstrating an inverse relationship between obesity and lower income brackets. In our sample, approximately one-third of the men residing in rural Norway were directly engaged in fishing or agricultural work compared to none of those residing in Oslo. The pattern of results for rural men with less income likely reflects variations in the working environment due to occupational type (e.g., heavy labor), which partially determines overall levels of energy expenditure.

Regarding education, our results also varied according to gender, and suggested that women with a lower level of education had a greater baseline BMI than their more educated counterparts. Indeed, education level is generally thought to affect a variety of obesity-related health behaviors, and one study of Norwegian adolescents demonstrated inverse associations between parental level of education and adolescent obesity [15]. However, this result had lost significance by the year 2001.

No significant findings existed between the socio-economic indicators and changes in BMI over time. Some have argued that social and economic gaps in BMI distribution may ultimately narrow, owing to blanket exposure to an increasingly obesogenic environment [14]. This stance potentially draws support from a Norwegian study of adult men, which reported a universal BMI increase across all educational levels over a 3-year period, such that men aged 40–42 years with less than 11 years education in 1994–1996 demonstrated a similar prevalence of obesity as those with 13–16 years of education in 1997–1999 [22].

Our study is limited by self-reported data, which may have produced an underestimate of weight and overestimate of height by participants [23]. However, published research on the accuracy of self-reported height and weight has shown high correlations (i.e., r's > .9) indicating the suitability of these data [24,25]. In addition, our cohort was representative of the cross-sectional population datasets upon which it was based, yielding similar BMI and prevalence estimates [26], thereby limiting the potential that selection or cohort bias affected the results. These findings also support existing research documenting trends of increasing obesity and overweight in Scandinavia. It is interesting to note that women had a lower BMI in our study than previous Norwegian estimates [1,22], although their BMI was similar to Swedish and Danish studies[3]. Strengths of the study involved the inclusion of two socio-economic indicators and continuous BMI was chosen as the primary measure of weight change. A direct investigation of occupational status on BMI status and weight change is a recommended area of future study, as it seemed plausible that type of occupation (e.g., farming/fishing) influenced results for men living in rural regions. Our sample had very few immigrants, which prohibited our ability to replicate previous findings from Norway showing disparity in obesity along ethnic lines [27].

## Conclusion

Globally, increases in population BMI are driving the epidemic of obesity. The significant and continued weight gain among persons already classified as overweight in 1990 is alarming. Owing to the notorious difficulties in treating obesity, early and increased awareness of overweight status may prove valuable in stimulating secondary prevention efforts. Research suggests a substantial proportion of adults do not have an accurate perception of their BMI status by incorrectly assuming their weight falls into a normal weight range, which is an erroneous assumption with serious implications for health behaviors [28]. Younger adulthood appeared to represent a critical time period at which accelerated weight gain occurred, likely due to ensuant lifestyle changes involving energy expenditure and dietary intake. Research on successful weight stability throughout young adulthood represents a valuable area of study to unlock key behavioral strategies successful in facilitating weight maintenance into later life. Targeting our interventions at critical lifetime transition periods may prove fruitful in limiting or reversing observed age-related trends.

## Competing interests

All authors, Deborah L. Reas, Jan F. Nygård, Elisabeth Svensson, Tom Sørensen, and Inger Sandanger, hereby declare that we have no competing interests.

## Authors' contributions

TS and IS conceived of the study, organized acquisition of data, and participated in the study design and drafting of the manuscript. DR, JN, and ES performed the statistical analyses, performed the review of the literature, and participated in drafting the structure, content, and organization of the manuscript. All authors revised it critically for intellectual content and gave approval of the final version.

## Pre-publication history

The pre-publication history for this paper can be accessed here:


